# Potential of Nutraceutical Supplementation in the Modulation of White and Brown Fat Tissues in Obesity-Associated Disorders: Role of Inflammatory Signalling

**DOI:** 10.3390/ijms22073351

**Published:** 2021-03-25

**Authors:** Federica Scarano, Micaela Gliozzi, Maria Caterina Zito, Lorenza Guarnieri, Cristina Carresi, Roberta Macrì, Saverio Nucera, Miriam Scicchitano, Francesca Bosco, Stefano Ruga, Anna Rita Coppoletta, Rocco Mollace, Jessica Maiuolo, Irene Bava, Antonio Cardamone, Monica Ragusa, Ernesto Palma, Vincenzo Musolino, Vincenzo Mollace

**Affiliations:** 1 Department of Health Sciences, Institute of Research for Food Safety & Health IRC-FSH, University “Magna Græcia” of Catanzaro, 88100 Catanzaro, Italy; federicascar87@gmail.com (F.S.); gliozzi@unicz.it (M.G.); mariacaterinazito@libero.it (M.C.Z.); lorenzacz808@gmail.com (L.G.); carresi@unicz.it (C.C.); robertamacri85@gmail.com (R.M.); saveri.nucera@hotmail.it (S.N.); miriam.scicchitano@hotmail.it (M.S.); boscofrancesca.bf@libero.it (F.B.); rugast1@gmail.com (S.R.); annarita.coppoletta@libero.it (A.R.C.); rocco.mollace@gmail.com (R.M.); jessicamaiuolo@virgilio.it (J.M.); irenebava@libero.it (I.B.); tony.c@outlook.it (A.C.); palma@unicz.it (E.P.); 2 Nutramed S.c.a.r.l., Complesso Ninì Barbieri, Roccelletta di Borgia, 88021 Catanzaro, Italy; 3 Department of Experimental and Clinical Medicine, University Magna Graecia of Catanzaro, 88100 Catanzaro, Italy; m.ragusa@unicz.it

**Keywords:** metabolic diseases, obesity, inflammation, WAT, BAT, browning, polyphenols, nutraceutical supplementation

## Abstract

The high incidence of obesity is associated with an increasing risk of several chronic diseases such as cardiovascular disease, type 2 diabetes and non-alcoholic fatty liver disease (NAFLD). Sustained obesity is characterized by a chronic and unsolved inflammation of adipose tissue, which leads to a greater expression of proinflammatory adipokines, excessive lipid storage and adipogenesis. The purpose of this review is to clarify how inflammatory mediators act during adipose tissue dysfunction in the development of insulin resistance and all obesity-associated diseases. In particular, we focused our attention on the role of inflammatory signaling in brown adipose tissue (BAT) thermogenic activity and the browning of white adipose tissue (WAT), which represent a relevant component of adipose alterations during obesity. Furthermore, we reported the most recent evidence in the literature on nutraceutical supplementation in the management of the adipose inflammatory state, and in particular on their potential effect on common inflammatory mediators and pathways, responsible for WAT and BAT dysfunction. Although further research is needed to demonstrate that targeting pro-inflammatory mediators improves adipose tissue dysfunction and activates thermogenesis in BAT and WAT browning during obesity, polyphenols supplementation could represent an innovative therapeutic strategy to prevent progression of obesity and obesity-related metabolic diseases.

## 1. Introduction

The prevalence of overweight and obesity is increasing worldwide, and it has reached epidemic proportions [1]. The World Health Organization (WHO) has estimated that over one billion of adult people are overweight and about 300 million are obese [2]. This represents a major risk factor for different diet-related chronic diseases, including cardiovascular disease, type 2 diabetes, hypertension, stroke, and non-alcoholic fatty liver disease (NAFLD). Traditionally, adipose tissue has been considered as a passive factor in the development of obesity as well as its consequences, which have a passive role [3]. In fact, physiologically, white adipose tissue (WAT) might be considered as a long-term deposit of triglycerides, from which free fatty acids (FFA) are released into the bloodstream, in case of insufficient energy intake, and used by other tissues (e.g., liver and skeletal muscle) as an alternative energy source [4]. Despite these consolidated evidence, recent perspectives show that WAT is considered a complex and dynamic endocrine organ capable of secreting a large number of adipose tissue-specific hormones, known as adipokines or adipocytokines, involved in several biological and physiological processes, such as inflammatory and immune processes, glucose and lipid metabolism, regulation of appetite, cardiovascular homeostasis and reproduction [5]. In obese patients, energy intake consistently exceeds energy expenditure, and adipose tissue is subject to different cellular and structural remodelling processes to balance the excessive caloric intake. Those processes include adipocyte hyperplasia (increase in cell number) and hypertrophy (increase in cell size), immune cells infiltration and extracellular matrix (ECM) remodelling, that are implicated in tissue expansion [6]. The hypertrophied adipocytes, in turn, may secrete adipokines that can directly alter insulin signaling and recruit macrophages, leading to inflammation in adipose tissue [7]. Sustained obesity, associated with chronic and unsolved inflammation, evolves into an imbalance of adaptive homeostatic mechanisms, leading to dysfunction of adipose tissue, with impaired secretion of adipokines, upregulation of the expression of proinflammatory adipokines, excessive lipid storage and adipogenesis as well as impaired angiogenesis, local hypoxia and fibrosis [8,9]. Unlike WAT, which stores energy, mammals, including humans, have brown adipose tissue (BAT), which is specialized for energy expenditure, a process known as thermogenesis, with a potential role against obesity and associated metabolic diseases [10]. Although chronic inflammation in adipose tissue is well defined, the related signals and the molecular mechanisms that trigger chronic inflammation are not well understood. The purpose of this review is to investigate and better clarify how inflammatory mediators act as signalling molecules during adipose tissue dysfunction in the development of insulin resistance and all obesity-associated diseases. In particular, little is known about the role of inflammatory signalling in BAT thermogenic activity and regarding the browning of WAT, which represent a relevant component of adipose alterations during obesity, as well as an interesting field of research for the identification of new therapeutic targets in the treatment of metabolic disorders. In this context, we have focused our attention on the most recent evidence in literature, with a particular regard on the nutraceutical approach in management of adipose inflammatory state, focusing on the common mediators leading to WAT and BAT dysfunction, with the aim of offering an overview in the prevention of progression of obesity-associated metabolic diseases.

## 2. Depot-Specific Differences in Infiltration of Adipose Macrophage and Role of Gut in Visceral Fat Inflammation

Development of techniques such as nuclear magnetic resonance and computed tomography led to the possibility to distinguish abdominal fat into subcutaneous and visceral one. In obese people inflammation is much more pronounced in visceral fat, with a higher accumulation of inflammatory adipose tissue macrophages (ATMs) and a greater secretion of proinflammatory cytokines, compared with subcutaneous fat [11,12]. Moreover, during lipolysis, FFAs secreted from visceral adipose tissue could reach directly the liver via portal vein, whereas FFAs from subcutaneous fat enter the peripheral circulation. Therefore, increased visceral fat could be an important contributing factor in hepatic steatosis development [13].

The collective term of “visceral fat” includes several fat depots in the peritoneal cavity, for instance, mesenteric, omental, and perirenal fat, which differ both structurally and metabolically from each other. In particular, different studies have shown a difference in the total number of macrophage and/or macrophage phenotypes between fat depots. The amount of infiltrating macrophages is approximately two- to fourfold higher in omental as compared to subcutaneous fat [14]. Likewise, in high-fat diet (HFD)-fed mice, an increase in macrophage infiltration in epididymal fat compared with subcutaneous fat was observed [15].

**Figure 1:**
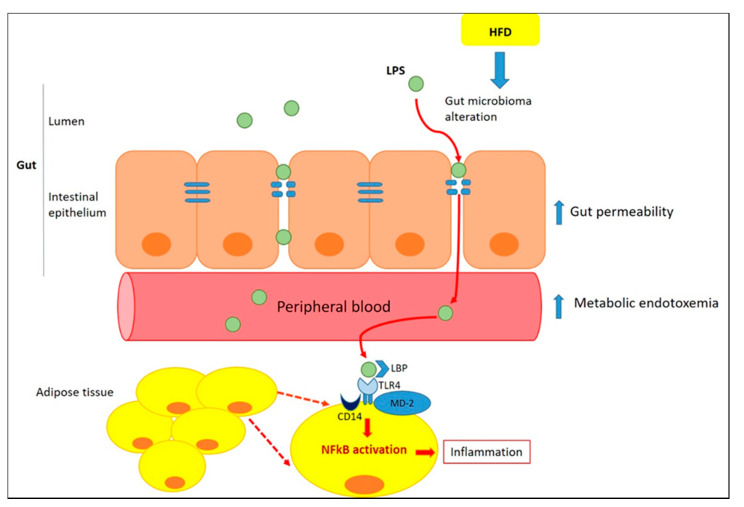
**Role of gut in inducing adipose tissue inflammation.** Inflammation alters the gut barrier function through a reduction in the thickness of the intestinal mucus layer and a subsequent increase in gut permeability [18]. Lipopolysaccharide (LPS), trigger inflammation in adipocytes and, along with macrophage colony-stimulating factor (M-CSF), infiltrated from the intestine, promotes ATM recruitment within the mesenteric fat depot [19]. Once in the circulation, LPS could reach adipose tissue and acts as a Toll-like receptor agonist (TLR)-4, increasing the expression of proinflammatory markers [20]. TLR4 binds to LPS through the formation of a complex with LPS-binding proteins (LBP), MD-2 (myeloid differentiation factor 2) and CD14 (cluster of differentiation 14). This complex activates the myeloid differentiation primary-response protein 88 (MyD88)-dependent pathway leading to Nuclear factor- κB (NF-κB) activation and to transcription of proinflammatory genes both in adipocytes and ATM [21,22].

The crosstalk between white adipocytes and WAT-infiltrating immune cells represents a central process in the physiopathology of obesity, although the mechanisms underlying the visceral-specific ATMs infiltration are unclear [16]. The key question should be sought in the causes of chronic low-grade inflammation and macrophage infiltration in adipose tissue. In recent years, a considerable attention to the involvement of gut dysfunction and microbiota profile alteration inducing adipose tissue inflammation has been paid [17] (Figure 1).

### 2.1. Inflammation of White Adipose Tissue (WAT)

Chronic, low inflammatory state of adipose tissue, observed in obesity, can derive both from external stimuli, such as LPS-derived from gut microbiota, and from different metabolic stressors. These stressors include fibrosis and mechanical stress resulting from rapid adipose tissue expansion and inadequate remodelling of extracellular matrix to accommodate adipocyte growth. Under condition of metabolic stress, the expression of Collagen alpha-3(VI) chain (col6a3) increased significantly in the epididymal fat of *ob/ob* and db/db mice (1.3-fold and 1.4-fold, respectively). The close relationship between abnormal collagen deposition and inflammation of adipose tissue was demonstrated by a trend towards reduction in F4/80 mRNA levels in epididymal fat and a significant reduction in mesenteric fat of col6KOob/ob mice compared to *ob/ob* mice [23]. Hypoxia represents another risk factor for the induction of chronic inflammation in adipose tissue. Ye et al. showed that hypoxia occurred in adipose tissue of *ob/ob* mice with a 70% of reduction in interstitial partial pressure of oxygen (P_O2_) and an increase in hypoxia-inducible factor (HIF)-1α mRNA levels in epididymal fat pad, compared to control mice. Moreover, in epididymal fat of *ob/ob* mice, hypoxia was associated with an up-regulation of inflammation-related genes, such as TNFα, IL-1, IL-6, CD11 and F4/80 [24].

**Figure 2:**
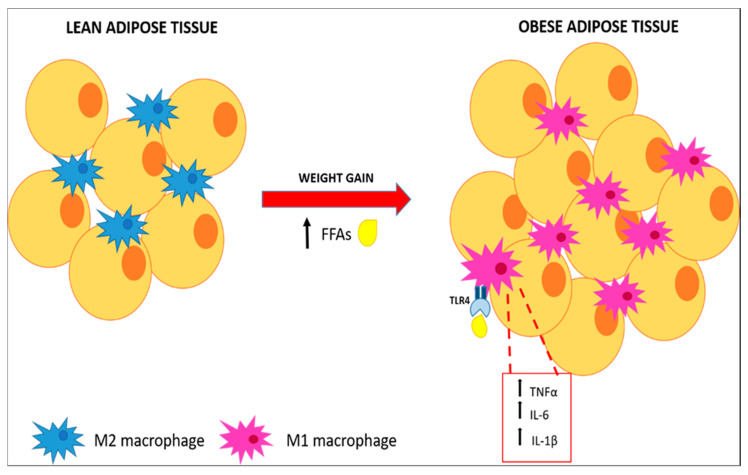
**Macrophages polarization and pro-inflammatory cytokines expression.** FAAs released by hypertrophic adipose tissue stimulate polarization of M2 macrophages through activation of the TLR4 pathway resulting in pro-inflammatory cytokines secretion.

In the WAT, the number of macrophages is directly correlated with adiposity and with adipocyte size in both human subjects and mice [25]. Recent data suggest that adipocytes can dedifferentiate into preadipocytes [26] and they could even differentiate to a multipotent cell population [27,28] Notably, adipocyte precursors and preadipocytes might efficiently differentiate into typical macrophages [29,30]. Nonetheless, experimental studies have shown, a clear generation of WAT macrophages within the bone marrow, indicating that macrophages present in adipose tissue do not derive from in situ preadipocytes differentiation, but rather from circulating monocytes that infiltrate WAT [31]. FFAs released by hypertrophic adipocytes, serve as a ligand for TLR4, inducing inflammation through NF-κB activation [32]. Activated TLR-4 changes the phenotype of macrophages from M2, a partially activated state, to M1, a constant activated state, which surround apoptotic adipocytes in a “crown-like” structure, secrete high levels of tumor necrosis factor α (TNFα), IL-6 and IL-1β, and enhance the inducible nitric oxide synthase (iNOS) expression (Figure 2). The direct influence of TLR4 activation on ATM polarization was observed in an in vivo study, in which HFD-induced TNFα overexpression was attenuated in TLR4^−/−^ mice. Furthermore, TLR4 deficiency increased mRNA expression of C-type lectin domain family 10 member A (Clec10a), mannose receptor C type 1 (Mrc1), and macrophage galactose N-acetyl-galactosamine specific lectin 2 (Mgl2), M2 markers [33].

Once secreted, TNFα binds adipocytes and provokes a cascade of intracellular reactions leading respectively to an increased lipolysis and to an increased expression of different pro-inflammatory genes, e.g., CC-chemokine ligand 2 (CCL2, also referred as monocyte chemotactic protein-1, MCP-1) [34]. Of particular note, in WAT of obese mice the increased mRNA levels of inflammation- and macrophage-related genes preceded an intense increase in insulin production [35]. Furthermore, treatment with rosiglitazone, which is known to increases systemic insulin sensitivity and reduces adipose tissue fatty acid efflux, probably through the activation of the nuclear Peroxisome Proliferator-Activated Receptor-γ (PPAR-γ), downregulated the expression of these pro-inflammatory genes [35]. Hotamisligil et al., hypothesized that TNFα represents a direct link between obesity-induced inflammation and impaired insulin sensitivity. In fact, in their study was demonstrated that adipose expression of TNFα mRNA was elevated in obese Zucker fa/fa rats. Administration of a soluble TNFα receptor lead to TNFα neutralization, improving insulin-resistance [36].

Thus, the temporal appearance of these inflammatory molecules before the development of insulin resistance, as well as their known ability to facilitate insulin resistance and other obesity-complications, strongly indicates inflammation of adipose tissue as a major player in the development of obesity-related complications [35].

### 2.2. Intracellular Pathways That Control Inflammatory Signaling

**Figure 3:**
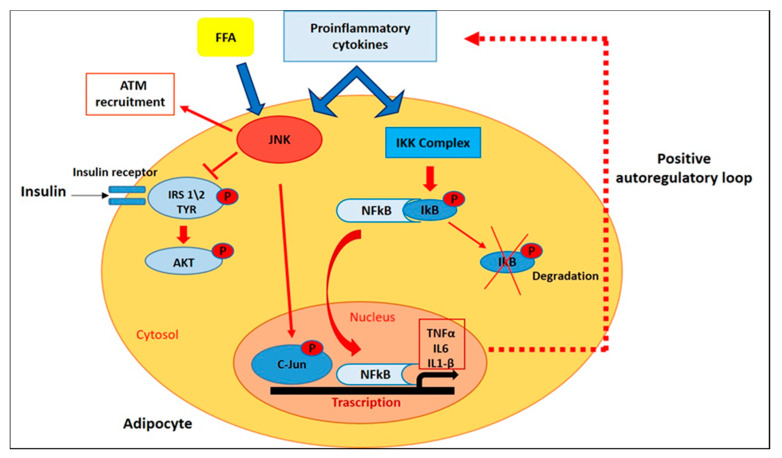
**Intracellular Pathways that Control Inflammatory Signaling.** In adipose tissue of obese patients, FFAs and proinflammatory cytokines mediate the activation of JNK and NF-kB pathways, which in turn promote insulin resistance and the transcription of target genes encoding for inflammatory mediators.

In adipose tissue of obese patients, many of the proinflammatory cytokines and adipokines produced in visceral WAT (vWAT), including TNFα, IL-1β, IL-6, and resistin, mediate the activation of the Jun N-terminal Kinases (JNKs) [37]. There are three different JNK isoforms (JNK-1,-2, and -3), which are members of the Mitogen-Activated Protein Kinases (MAPK) family. JNK-1 and JNK-2 are ubiquitously expressed, while JNK-3 expression is restricted to the brain, testis, and pancreatic β-cells. They are induced in response to cellular stress signals and are able to phosphorylate and activate the protein c-Jun, a member of the Activator Protein-1 (AP-1) transcription factor family [38]. For explaining the JNKs-role in the induction of insulin resistance in response to excess of adiposity, different mechanisms were proposed. First of all, JNKs stimulated by increasedlevels of TNFα and FFAs, are responsible for the phosphorylation of insulin receptor substrate (IRS-1) in serine-307 (Ser-307), inhibiting the interaction of IRS-1 with the insulin receptor and tyrosine phosphorylation of IRS-1 in response to insulin [39]; in fact, no increase in phosphorylation of IRS-1 at Ser-307 was detected through immunoblot analisys of liver tissue of obese Jnk1^−/−^ mice, demonstrating that Ser-307 of IRS-1 is a target for JNK action in vivo [40,41]. Furthermore, JNK1 and 2 mediate the recruitment and activation of macrofages in vWAT; in fact, HFD-fed mice with a macrophage-specific ablation of JNK exhibited a less severe insulin resistance and were largely protected from the inflammation associated with diet-induced obesity, through a decreased accumulation of ATM and a reduced expression of genes associated with M1 polarization [42,43] (Figure 3). In addition to negative interaction of JNK with IRS-1 signaling in insulin-sensitive peripheral tissues, this kinase in a transgenic mouse model (MKK7D) has been shown to promote insulin-resistance by acting in pancreatic *β*-cells, through inhibition of insulin secretion and insulin-induced Akt phosphorylation and reducing the expression of downstream insulin-target genes [44]. JNK inhibition reduces the release of IR-related pro-inflammatory cytokines such as TNFα and MCP-1 and represents an attractive opportunity to improve metabolic dysfunction in obesity by preventing excessive adiposity, insulin resistance and inflammation [45].

Along with JNK-activation, IKK\NF-κB signalling pathway plays critical roles in inflammation, immunity, differentiation, cell proliferation, and survival [46]. After stimulation with different pathogenic stimuli, such as cytokines and pathogen-associated molecular patterns (PAMPs), NF-κB activation promotes the transcription of target genes encoding for inflammatory mediators such as TNFα, IL-6, and IL-1β [45]. In white adipose tissue, NFκB activation was recently demonstrated in transgenic mice engineered with a luciferase construct driven by an NFκB-responsive promoter (HLL mice) [47]. After luciferin injection, HFD-fed HLL mice demonstrated a 2-fold increase in abdominal luminescence compared to control mice, and a 5-fold NF-κB transgene activation in subcutaneous and visceral adipose tissue. Furthermore, in eWAT from HFD-fed mice, immunofluorescence analysis showed increased expression of NFκB (RelA/p65) in the nuclei of F4/80^+^ ATM clusters [48]. In the same study, PCR analysis showed that HFD induces a significant increase in the mRNA expression of the inducible IKK family member IKKɛ in WAT, while mice bearing a targeted deletion of IKKɛ are surprisingly protected from diet-induced obesity and adipose inflammation [48].

Growing evidence indicates that TNFα is a major mediator of inflammation in general, of insulin resistance and of obesity in particular. In fact, it’s known that TNFα is overexpressed in adipose tissue of genetically obese mice and its levels is correlated with C-reactive protein (CRP) levels, a marker of systemic inflammation [36,49]. In addition, TNFα induces insulin resistance through serine phosphorylation of IRS-1, with consequent impairment of normal insulin signaling [50]. Many of the TNFα-mediated effects are expressed by the activation of NF-κB, and the expression of TNFα itself is regulated by NF-κB [51,52], thus establishing a positive autoregulatory loop that may intensify the inflammatory response and the duration of chronic inflammation (Figure 3). Tourniaire et al. showed that treatment with TNFα (15 ng/mL) for 24h in primary cultures of human adipocyte resulted in an increased chemokines mRNA expression, in particular CCL5, CCL19 and CCL20. Then, to corfirm the involvement of NF-κB in the regulation of chemokine expression in WAT, two transgenic models were used: aP2-p65 overexpressing mice and NF-κB p65 null mouse embryonic fibroblasts (MEFs). qPCR analysis showed in WAT of aP2-p65 mice a 40-fold increase of CCL5 and CCL19, while a basal chemokine expression in untreated cells was always lower in p65 null MEFs than in wild-type (WT) cells. On the other hand, TNFα treatment of WT MEF cells resulted in a 3.5-fold upregulation of TNFα, IL6 and chemokine mRNA expression, while a no statistically significant induction was observed in p65 null cells in response to TNF-α, confirming that NF-κB is the main mediator of chemokine expression in response to TNF-α [53]. In another study, the treatment of human subcutaneous WAT (sWAT) for 18h with different concentrations of two NF-kB inhibitors, sulfasalazine and BAY 11-7082, significantly inhibited the release of IL-6, IL-8, TNFα and NFkB p65 DNA-binding activity. Furthermore, both sulfasalazine and BAY 11-7082, at the highest concentrations, reduced IKKβ and increased insulin receptor-β protein expression in adipose tissue. These data suggest that in adipose tissue, the control of the IKKβ/NFkB pathway could provide a therapeutic strategy for regulating pro-inflammatory cytokines release and thereby alleviating insulin resistance [54].

### 2.3. Involvement of Endoplasmic Reticulum in WAT Inflammation

Hypertrophic growth of the adipose tissue is due to the high circulating levels of lipids and their accumulation in an unilocular cytoplasm represents an adaptive response to overnutrition, essential for maintaining systemic energy homeostasis [55]. Kim et al. showed that differentiated 3T3-L1 adipocytes treated for 6 days with saturated fatty acids (SFAs) and a monounsaturated fatty acid (MUFA), became hypertrophic in time- and dose-dependent manners, and Oil red O staining and scanning electron microscopy analysis revealed a cytoplasm containing enlarged uniloculus-like lipid droplets [56]. Another alteration in hypertrophic adipocytes is the accumulation of radical oxygen species (ROS) and endoplasmic reticulum (ER) stress [57,58].

In adipose tissue, ER stress can originate through accumulation of unfolded proteins, excess of nutrients and FFAs, glucose deprivation due to insulin resistance, and decreased vascularization, that can lead to the consequent activation of an adaptive response known as unfolded protein response (UPR) [43]. ER Stress and UPR are linked to major inflammatory and stress signaling pathways with a central role in obesity-induced inflammation and metabolic dysfunction, via activation of JNK1 and IKK-NF-κB pathways, as well as ROS production [59]. In adult-derived human adipocyte stem (ADHAS) cells, ER stress induction with different ER stress-inducing agents such as tunicamycin (glycosylation inhibitor), thapsigargin (Ca^2+^-ATPase inhibitor), or palmitate (saturated fatty acid), has been shown to trigger an inflammatory response, as resulted by an increase in TNFα mRNA expression and a decrease in Iκβ-α, indicative of NF-κB activation [60].

The role of ER stress and its inflammatory response was also investigated in epididymal adipose tissue of HFD-fed mice. After 16 weeks of hyperlipidemic diet a significant up-regulation of mRNA levels of ER markers, such as Bip, c/EBP-homologous protein (Chop), ER degradation enhancer mannosidase (Edem), activating transcription factor 4 (Atf4), protein disulfide isomerase (Pdi) and ER DnaJ homolog 4 (Erdj4) has been shown [61]. Moreover, the link between obesity-induced ER stress and chronic inflammation was confirmed by a notably increase in TNFα mRNA levels and macrophages infiltration in adipose tissue of obese mice [61]. In another study, it was found that 4-phenyl butyric acid (PBA) treatment, a chaperone known to improve ER folding capacity, in leptin-deficient (*ob/ob*) mice resulted in a reduction of PERK and IRE-1α phosphorylation in adipose tissue, compared to vehicle-treated group. Furthermore, this effect was associated with a reduction of JNK activation and IRS-1 phosphorylation at Ser-307, indicative that in adipose tissue, the improvement of ER stress could be associated with an increase in insulin action [62].

**Figure 4:**
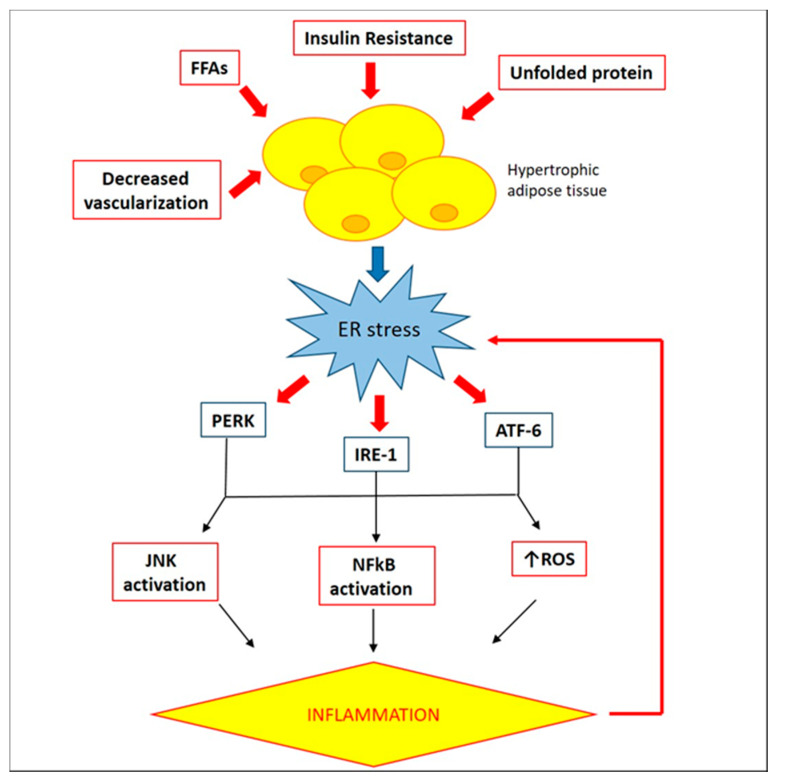
**Involvement of ER stress in adipose tissue inflammation.** In obese adipose tissue, accumulation of unfolded proteins, excess of FFAs, insulin resistance, and decreased vascularization determine ER stress, responsible of an inflammatory response via activation of JNK and NF-κB pathways, as well as production of ROS.

In adipose tissue, UPR signalling represents an inducer of ER stress that synergizes with other molecular mechanisms inducing inflammation (Figure 4). Indeed, TNFα was shown to activate the UPR in mouse fibrosarcoma cells through a mechanism that involves ROS production via NADPH oxidase activation [63]. This points out oxidative stress as another process linked to obesity-induced ER stress, as confirmed by a significantly reduction of Bip and CHOP mRNA level in 3T3-L1 adipocytes pre-treated with N-acetyl cysteine (NAC), a ROS inhibitors [61].

In addition, in hypertrophic adipose tissue, adipokines- and FFAs-induced ROS overproduction is associated with a reduction of antioxidant defenses, such as serum superoxide dismutase (SOD) and catalase (CAT). In WAT of diabetic obese KKAy mice, increased expression of NADPH oxidase mRNA was associated with a decrease in cytoplasmic SOD, CAT and glutathione peroxidase (GPx) mRNA levels, compared to control group [57,64].

## 3. Inflammation of Brown/Beige Adipose Tissue

Brown adipose tissue (BAT) compared to WAT seems to exert an opposite role. BAT includes brown adipocytes and the stroma vascular fraction (SVF), which contains several cells, such as pre-adipocytes, stem progenitor cells, and immune cells [65]. Brown adipocyte has a polygonal form with a central nucleus in a large cytoplasm containing small lipid droplets, a primitive RE and numerous mitochondria [66]. Moreover, BAT is highly innervated and vascularized [65]. Originally, BAT was thought to be present in humans only during the neonatal period [67]. Recently, considerable depots of brown adipocytes were recognised by positron emission tomography (PET) in cervical, paravertebral, supraclavicular, para-aortic, mediastinal, and adrenal regions in adult human [68].

While WAT mainly accumulates excess energy in the form of TAGs (triacylglycerols), BAT is specialized in energy dissipation in the form of heat through uncoupling protein 1 (UCP1)-mediated uncoupling of oxidative phosphorylation from ATP synthesis [69]. This process, mediated by temperature and diet, is known as non-shivering thermogenesis, and may protect against obesity by promoting energy expenditure [70].

In conditions of enhanced adaptive energy expenditure, cells which are similar to brown adipocytes appear in WAT, especially within the sWAT depots. This phenomenon is called “*browning*” of WAT, and inducible brown adipocytes developed during this process are called “beige” or “brite” (from “brown-in-white”) [71]. In contrast, the brown adipocytes developing in WAT during browning process, derived from Myf5-negative cells, that more closely resemble white adipocyte precursors [72].

Seale et al. showed that PR domain 16 (PRDM16) represents a key regulator that influences the cellular phenotype of brown fat. Indeed, it promotes brain fat cell character before and/or during adipogenic differentiation. In addition, transgenic expression of PRDM16 in eWAT induced an increase in the mRNA levels of UCP1, peroxisome proliferator-activated receptor gamma coactivator 1-alpha (PGC-1α) [73].

As discussed previously, several studies have focused on the role of WAT inflammation in obesity and obesity-associated systemic metabolic disfuction (e.g., insulin resistance); while little is known about the role of inflammatory signalling in BAT thermogenic activity and browning of WAT. We will therefore highlight the common mediators of inflammation that link the dysfunction of the two types of adipose tissue on the basis of the most recent evidence in the literature.

Compared to WAT, BAT is more resistant to developing local inflammation in response to mild obesity. This has been suggested by Fitzgibbons et al. study showing that BAT of mice fed a HFD for 13 weeks was more resistant than WAT to macrophage infiltration, as demonstrated by little or no F4/80 immunostaining of BAT sections [74]. The onset of BAT inflammation in obese mice seems to appear in a time-related way. Indeed, the upregulation of proinfammatory genes (TNFα, IL-6, MCP-1 and IL-1β) in interscapular BAT, becomes quite pronounced when mice have been fed HFD for 24 weeks [75]. This suggests that the inflammatory response in BAT is slower than in WAT, but increases where the obesogenic conditions are maintained.

**Figure 5:**
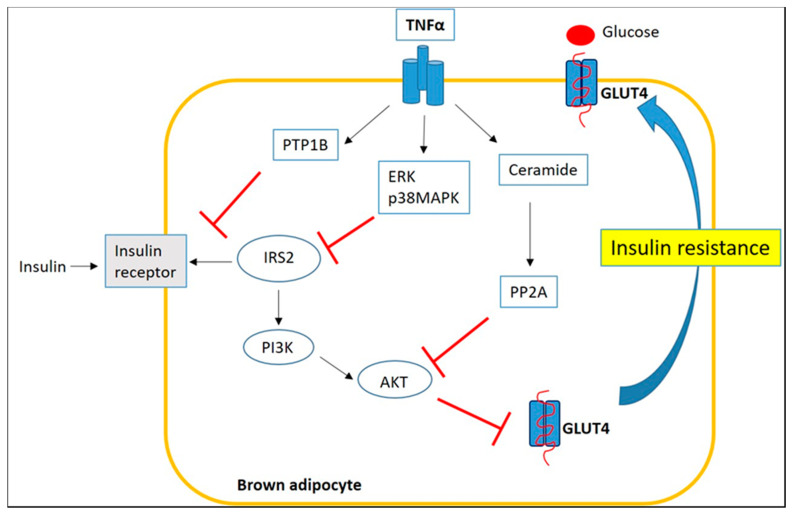
**Inflammatory signaling impairs insulin sensitivity of BAT through a TNFα-mediated mechanism**.

Therefore, we highlight how inflammatory environment of obese BAT affects its function and thermogenesis process. First of all, glucose uptake is essential for the thermogenesis process and, in a state of obesity, pro-inflammatory signaling can alter BAT insulin sensitivity through a complex TNFα-mediated mechanism. The mechanisms underlying this process involve: (1) Ser/Thr phosphorylation of the IRS-2 by p38MAPK and ERK with consequent impairment of IRS2-associated PI3K activity; (2) formation of ceramide and activation of the phosphatase PP2A, keeping AKT in an inactive dephosphorylated state; and (3) increased protein-tyrosine phosphatase 1B (PTP1B) activity, a negative regulator of insulin signaling, which dephosphorylates tyrosine residues of the insulin receptor (IR) and IRS-1 [76,77] (Figure 5).

In addition to their effects on insulin-sensitivity, pro-inflammatory cytokines appear to alter the specific thermogenic activity of BAT and WAT browning.

An in vitro study reported that TNFα derived from activated RAW 264.7 macrophages suppressed the induction of UCP1 mRNA expression level in C3H10T1/2 (10T1/2) adipocytes, differentiated from mesenchymal stem cells. ERK activation was also highlighted in this study as a possible mechanism by which TNFα suppresses UCP1 expression in obese white adipocytes [78]. Furthermore different in vivo studies have shown that, during an obesogenic diet, macrophage-derived pro-inflammatory cytokines (mainly TNFα and IL-1β) suppress the expression of UCP1 gene in BAT and WAT [79,80]. In addition, TNFα activity has been shown to induce apoptosis of brown adipocytes and an alteration of multilocular morphology of adipocytes [81]. Indeed, in genetically obese (*ob*/*ob*) mice with TNFα signaling deficiency, TUNEL staining showed that in BAT the number of apoptotic nuclei were significantly lower than those observed in obese *(ob/ob*) controls, as well as multilocular brown adipocytes were more abundant than in *ob/ob* mice [81].

As described above, LPS- and FFA-mediated TLR4 activation in WAT plays a key role in activating pro-inflammatory signaling and thus obesity-dependent insulin resistance [82]. More recently, activation of TLR4 by LPS or HFD has also been found to impair cold-induced WAT browning and thermogenesis. Indeed, it has been shown that LPS-injected mice show a significant increase in TLR4 mRNA expression compared with saline controls. Furthermore, LPS stimulation attenuates UCP1 protein levels as well as cold-induced brown-specific hallmark gene expression of UCP1, PGC-1α, PRDM16, and cell death-inducing DFFA-like effector A (CIDEA). Finally, TLR4-mediated defective thermogenesis has been associated to ER stress, as demonstrated by an increase of ER stress markers such as CHOP, GRP78/BiP (ER chaperone), and JNK phosphorylation [83]. Likewise, the thermogenic activity of BAT is sensitive to pro-inflammatory signaling. TLR4/TLR2 activation in murine brown fat cells has been shown to induce a pro-inflammatory response through activation of MAPK and NF-kB signaling pathways, resulting in decreased UCP1 expression and suppression of mitochondrial respiration [84].

## 4. Targeting Adipose Tissue Inflammation to Treat Obesity and Obesity-Related Complications

The development of therapeutic strategies targeting adipose tissue dysfunction might be also useful in the prevention of metabolic disorders, typically associated with obesity. Several anti-obesity drugs have been approved by the Food and Drug Administration (FDA) and by the European Medicines Agency (EMA) for long-term treatment of obesity, such as orlistat, lorcaserin, phentermine-topiramate, naltrexone-bupropion, and liraglutide [85]. Among them, noradrenergic sympathomimetic drugs such as phentermine, diethylpropion, phendimetrazine act as appetite suppressor and are used for less than 12 weeks in the management of obesity. These drugs, however, are known for their unpleasant side-effects such as anxiety, dizziness, headache, elevated heart rate and gastro-intestinal outcome [86]. Another anti-obesity remedy is Orlistat. It is a powerful inhibitor of gastrointestinal lipase, an enzyme that acts by hydrolysing dietary fats in the form of triglycerides, thus reducing the subsequent absorption of monoglycerides and free fatty acids and favoring dietary fat excretion of up to 30%. However, this mechanism of action can determine unpleasant gastrointestinal side effects related to fat malabsorption [86].

Among the few approved anti-inflammatory treatments for counteracting obesity, weight loss, dietary intervention and lifestyle modification represent the golden target [87,88,89]. This is also confirmed by studies conducted in diet-obese mice which have shown that weight loss is associated with a reduction in adiposity accompanied by a decrease in macrophage infiltration of adipose tissue as well as gene expression of pro-inflammatory markers [89,90].

In this context, nutraceutical supplementation might represent a valid approach aimed to ameliorate WAT inflammation avoiding the typical side effects of drug therapy against obesity.

### 4.1. Effects of n-3 PUFA on Adipose Tissue Inflammation and BAT Function

Long-chain *n*-3 PUFA are essential fatty acids that the human body is unable to synthesize and must be consumed through the diet, in particular from marine sources (especially fatty fish and fish oil supplements). Eicosapentaenoic acid (EPA) and docosahexaenoic acid (DHA), that are the most represented *n*-3 PUFA, have shown anti-inflammatory properties and may have a significant impact on chronic inflammation, including obesity related inflammatory diseases [91]. EPA and DHA exert their activity through a PPARα/γ agonism and inhibition of NF-κB binding activity, suggesting a direct mechanism of *n*-3 PUFAs in the regulation of target genes and anti-inflammatory effects. Moreover, *n*-3 PUFAs can activate PPARα thereby increasing expression of fatty acid oxidation genes and resulting in a reduction in hepatic and plasma triglycerides [92,93]. Another mechanism by which EPA and DHA stimulate fatty acid oxidation is through AMP-activated protein kinase (AMPK)-activation in adipose tissue and cultured adipocytes [94]. EPA and DHA increase plasma adiponectin secretion from adipose tissue in obese humans and rodents, improving insulin sensitivity. This effect on adiponectin secretion is PPARγ-dependent, in fact it has been shown that adiponectin levels are not elevated in fish oil-fedding mice lacking PPARγ [95]. They also induce leptin secretion, that reduces energy intake and increases energy expenditure, and visfatin secretion, that shows an insulin-mimetic action. Furthermore, in adipose tissue, *n*-3 PUFA lead to a marked decrease of M1 macrophage accumulation and to the reduced expression of different pro-inflammatory cytokines, such as TNFα, MCP-1, IL-6, and PAI-1, through binding to G protein-coupled receptor 120 (GPR120), and consequent inhibition of NF-kB and JNK pathways. The protective effect of this receptor, present in both adipocytes and macrophages, is confirmed by the absence of EPA-mediated improvement on insulin sensitivity in GPR120 null mice [96,97,98]. Compared to WAT, there is far less information on *n*-3 PUFAs effects on BAT and beige adipocytes. Nevertheless, in recent years, different studies have shown beneficial results of *n*-3 PUFAs on BAT thermogenic activity and WAT browning [99]. In addition to WAT, GPR120 is highly expressed in BAT. Stimulation of GPR120 signal results in intracellular Ca^2+^ release, mitochondrial depolarization and fragmentation, which happen together with mitochondrial UCP1 activation, to increase synergistically mitochondrial respiration. Consequently, GPR120 stimulation in BAT determines an increase in fat oxidation and decrease in brown adipocyte lipid content [100]. Moreover, a recent study showed that *n*-3 PUFA and synthetic agonists-mediated GPR120 activation, induce a significant upregulation of brown-related thermogenesis markers in BAT and WAT browning, via the release of hormonal factor fibroblast growth factor-21 (FGF21) [101].

To obtain substantial and beneficial GPR-mediated anti-inflammatory effects, large amount of *n*-3 PUFA should be consumed, thus, a high-affinity, small-molecule GPR120 agonist (cpdA) was proposed as a potential clinical alternative to fish oil treatment. In vivo study showed that cpdA administration to obese mice exerts potent anti-inflammatory and insulin-sensitizing effects, with a decreased of hepatic steatosis. These data should be confirmed in human clinical trials, as they are also needed to characterize the potential benefit of *n*-3 PUFA supplementation on activation of brown/beige adipose tissue [102,103]. In fact, while different meta-analyses have shown protective effects of *n*-3 PUFA in diseases ranging from cardiovascular diseases to inflammatory conditions, other clinical studies have not observed beneficial effects on these endpoints [104,105]. The disparity in reported benefits of *n*-3 PUFA may derive from the doses used and the high noncompliance rate, the duration of treatment, the source of chosen *n*-3 PUFA.

### 4.2. Role of Dietary Polyphenols on Adipose Tissue Inflammation

Polyphenols, such as stilbenes, flavonoids and curcuminoids, are natural compounds largely presents in fruits, vegetables, cereals and beverages with a broad range of biological functions such as antioxidant, anti-inflammatory, anti-obesity, anti-thrombotic, anti-aging and anti-cancer, which afford potential efficacy in modulation of many pathological conditions, in particular those caused by oxidative stress and/or chronic inflammation, such as cardiovascular diseases (CVD) and metabolic diseases [106,107]. Here, we highlight how dietary polyphenols exert their beneficial effects against adipose tissue inflammation and in particular we will analyse if polyphenols have a positive impact on WAT browning and BAT thermogenic activity through the modulation of inflammatory signalling.

#### 4.2.1. Resveratrol (RSV)

Resveratrol (RSV) (trans-3,5,4′-trihydroxystilbene) is a polyphenolic compound mainly contained in red grapes, red wine, mulberries, peanuts and cocoa. Its chemical structure consists of two phenolic rings linked together by a styrene double bond which results in two isomers (cis and trans), with the trans-isomer as the most stable and biologically active [108]. Approximately, 75% of RSV is absorbed after its oral administration in humans. However, most is metabolised in the intestine and liver as glucuronides and sulfates metabolites, thus reducing its bioavailability [109]. It possesses a broad range of biological activities, including anti-inflammatory, vasoprotective (both cerebral and peripheral), antioxidant and insulin-sensitizing properties [110]. In obese mice, RSV showed a considerably capacity to reduce adipose tissue and total body fat, through its ability to increase the phosphorylation and AMPK activation, resulting in up-regulation of fatty acid oxidation, suppression of hepatic gluconeogenesis, and an increase in glucose uptake through translocation of GLUT4 [111,112]. The beneficial effects of RSV on adipose tissue inflammation result from its ability to suppress NF-κB and ERK activation. In particular, it was shown that RSV was able to inhibit NF-κB nuclear translocation in the adipocytes stimulated by TNFα and to prevent degradation of IκB [113]. In agreement with this in vitro study, RSV diet supplementation (0,4%) attenuated up-regulation of pro-inflammatory cytokines TNFα, interferon α and β (IFNα, IFNβ) and IL-6, as well as their upstream signaling molecules, including TLR2/4, MyD88, toll interleukin 1 receptor (TIR) domain containing adaptor protein (Tirap), TIR-domain-containing adapter-inducing interferon (TRIF), TNF receptor associated factor 6 (TRAF6), interferon regulatory factor (IRF5), p-IRF3, and NF-κB in the epididymal adipose tissues of HFD-fed mice for 10 weeks. These results were associated with reduction of FFA plasma concentration, known ligand of TLR2 and TLR4, suggesting that RSV may improve obesity-induced inflammation by both down-regulating TLR2 and TLR4 expression in epididymal adipose tissue and reducing plasma FFA levels in mice receiving a HFD [114]. Furthermore, it has been shown that in differentiated human adipocytes in primary culture, stimulated with IL-1β, RVS carries out its anti-inflammatory response via a SIRT1-dependent mechanism, increasing adiponectin levels and reducing inflammatory cytokines expression and secretion [115]. Recently, it has been shown that RSV is also able to modulate the thermogenic process, by SIRT1 activation, with an enhancement of the gene expression of UCP1 following LPS or IL-1β treatment in either in vivo or in vitro experiments [116]. Another in vivo study showed that RSV induced WAT browning in KKAy mice treated with control diet enriched with or without several doses of RSV for 12 weeks. This event was associated with an increase of browning gene expression levels (UCP1, PRDM16, FNDC5, TMEM26, and CIDEA) in dose-dependent manner, associated to an up-regulation of SIRT1 and PRDM16 protein levels. Comparable results were observed in C57BL/6J mice fed HFD with or without RSV. These data show a reproducible positive effect of RSV, both in a spontaneous and HFD-induced obese mouse model, against obesity through the switching of adipose tissue from the “white” to “brown” phenotype. Furthermore, SIRT1 knockdown completely regained the RSV-elevated PRDM16 protein, as well as the RSV-increased CIDEA and TMEM26 mRNA, suggesting the indispensable and primary role of SIRT1 in RSV-mediated regulation of metabolic syndrome. Interestingly, SIRT1-knockdown significantly reduced RSV-induced AMPK phosphorylation, showing that SIRT1 is the upstream factor of AMPK in the control of lipid metabolism and WAT browning [117,118]. Another interesting anti-obesity mechanism of RSV recently highlighted is due to its ability to improve the composition of gut microbiota in HFD-fed mice transplanted with RSV microbiota, with inhibition of pathogenic bacteria and stimulation of beneficial bacteria. This RSV probiotic effect reduces intestinal inflammation and restores the integrity of the intestinal barrier, which prevents LPS flux into the systemic circulation and activation of adipose tissue inflammation, as described above [119].

Based on this evidence, RSV represents a beneficial dietary bioactive molecule that can be easily incorporated into the diet to modulate adipose tissue inflammation and obesity-associated metabolic diseases, while more studies are needed to optimise its bioavailability before it can advance into clinical application.

#### 4.2.2. Curcumin

Curcumin is the most studied of all curcuminoids, is present in the spice turmeric and known for its obesity-associated anti-inflammatory, antioxidant, anti-angiogenesis, and chemotherapeutic features [120]. In HFD-induced obese mice, treatment with curcumin reduced NF-κB activity in liver tissue, with a consequent decreasing of hepatic inflammatory molecules. This effect was associated with a significant reduction in macrophage infiltration and an improvement in adiponectin expression in adipose tissue, and with higher circulating adiponectin levels. The mechanism underling these protective effects was correlated to curcumin’s suppression of IκB degradation and consequently inhibition of nuclear NF-κB translocation, resulting in reduced TNFα, IL-1β, IL-6 and COX-2 gene expression in differentiated adipocytes. Moreover, curcumin treatment reduced ER stress and increased SIRT1-mediated adiponectin transcription in adipocytes by activating forkhead transcription factor O1 (Foxo1) and enhancing Foxo1 and CCAAT/enhancer-binding protein-α (C/EBPα) interaction [121,122]. More recently, another HFD mouse model showed that curcumin treatment attenuated lipogenic SREBP-1c and ChREBP gene expression in hepatocytes, and blocked inflammatory response in the epididymal adipose tissue through inhibition of NF-kB expression, both in whole cell lysates and nuclear extract, and JNK signaling pathway activation [123]. In another in vivo study, 12 weeks of curcumin supplementation in HFD-fed mice decreased not only WAT macrophage infiltration, but also the degree of WAT M1-like macrophages compared to M2-like macrophages. Furthermore, intervention with curcumin was observed to increase CO_2_ production, O_2_ consumption, and energy expenditure, in HFD-mice undergoing metabolic cage analyses. This result was associated with an up-regulation of UCP1 levels in mouse BAT, via PPARα and PPARγ activation, without changes in UCP1 expression in subcutaneous inguinal adipose tissues [124].

In humans, curcumin is safe, even at high dosages (12 g/day), but it is known for its chemical instability at intestinal pH, water insolubility, bad oral bioavailability and high rate of urine excretion as glucuronide or sulfate [125]. For all these aspects, in a recent clinical trial, curcumin has been tested in obese patients affected from metabolic syndrome, in the form of phytosome and in presence of piperine [126]. The phytosome technology has stabilized curcumin and makes it highly bioavailable, while the presence of piperine has reduced the curcumin metabolism and excretion process [125,127]. This randomized, controlled study demonstrates that curcumin, associated with lifestyle intervention, was able to increase weight loss, enhance percentage reduction of body fat and increase waistline reduction [126]. Therefore, the previously mentioned anti-adipogenic and anti-inflammatory properties of curcumin in adipose tissue suggest its potential future application in bioavailable form in management of adipose tissue expansion and inflammation.

#### 4.2.3. Role of Citrus Flavonoids on Adipose Tissue Inflammation

Citrus fruits, such as bergamots, grapefruit, limes, lemons, mandarins, oranges, and pomelos are a considerable source of dietary flavonoids, in particular flavanones, flavones, flavonols and anthocyanins. The main flavonoids are the flavanones, such as neohesperidosides (naringin, neohesperidin and neoeriocitrin); and rutinosides (hesperidin, narirutin and didymin) [128]. The flavonoids present in citrus species are known for their antioxidant, cardiovascular protection and anti-inflammatory properties [129]. Several in vitro and in vivo studies have supplied strong evidence to support the protective effect of citrus flavonoids against adipose tissue inflammation associated with obesity. In an in vitro study, naringenin and hesperetin have shown their ability to inhibit TNFα-stimulated FFA secretion both in 3T3-L1 adipocytes and in mouse epididymal primary adipocytes, through the inhibition of NF-κB and ERK pathways. ERK pathway inhibition prevents TNFα from decreasing the transcription of two antilipolytic genes, perilipin and phosphodiesterase 3B (PDE3B); while, NF-kB pathway inhibition suppresses the transcription of IL-6, which also induces FFA secretion [130]. Moreover, it was shown that naringenin treatment, during adipocyte differentiation, significantly inhibited TLR2 mRNA expression, without affecting TLR4 mRNA expression, via PPARγ activation. Furthermore, naringenin inhibits TNFα-induced TLR2 mRNA expression by suppressing the NF-κB and JNK pathways in differentiated adipocytes, both in vitro and in vivo experiments, [131]. In addition, naringenin treatment for 14 days, suppresses HFD-induced macrophage infiltration into epididymal adipose tissue, as demonstrated by a significant reduction in Mac-2 mRNA expression and Mac-2 positive cells in immunohistochemical analysis. This effect was mediated by the suppression of MCP-1 expression, through inhibition of HFD-induced JNK phosphorylation in adipose tissue [132]. Anyway, little is known about the naringenin effects on WAT browning and BAT thermogenic activity; in particular, it would be interesting to investigate whether the anti-inflammatory activity of naringenin has a modulatory effect on the expression of UCP1.

*Citrus* derived polyphenols, such as naringin, neoesperidin and neoeriocitrin, as well as brutieridine and melitidine are particularly abundant in *Citrus bergamia* Risso et Poiteau. Recently, an enriched polyphenolic formulation, obtained from juice and albedo of Bergamot, and named Bergamot Polyphenolic Fraction (BPF) is known to have a lipid-lowering properties due several mechanisms of action, such as the increase of fecal neutral sterols and the total excretion of bile acids [133,134,135], the reduction of cholesterol absorption, via inhibition of pancreatic cholesterol ester hydrolase (pCEH) [135], and a statin-like action, due to the presence of brutieridin and melitidin, which are structural analogues of statins [136,137]. Recently, it has been reported that BPF phytocomplex exerts an anti-inflammatory activity in a diet-induced animal model of NAFLD, which reproducibly develops obesity, systemic inflammation, with crown-like bodies in adipose tissue, hypoadiponectinemia, increased IL-6 and TNFα, insulin resistance, dyslipidemia, progressing to NASH and fibrosis over time [138]. In particular, at the molecular level, BPF action can be attributed to the neutralization of free radicals and inhibition of JNK/p38 MAPK pathways, which have connected each other [139]. Due to its anti-inflammatory activity and its high flavonoids content, it could be hypothesized that BPF, through the modulation of inflammatory signalling, can prevent adipose tissue dysfunction associated with obesity, but further studies are needed.

Nobiletin (NOB) is a polymethoxylated flavone, present in some citrus fruits such as *Citrus depressa Hayata* (shiikuwasa) and *Citrus sinensis* L. (oranges), which for its anti-inflammatory activity has shown to regulate adipose tissue function in HFD-induced obese mice treated with NOB for 5 weeks. NOB treatment reduced significantly body and total WAT weight, plasma TGs and increased plasma adiponectin levels compared to HFD group. Furthermore NOB treatment increased energy expenditure related genes in WAT and improved insulin signalling through the inhibition of the inflammatory cytokines TNF-α and ikk/NF-κB activation [140].

Quercetin, is another flavonoids present in citrus fruits that could improve obesity-associated inflammation acting on multiple targets. Firstly, quercetin has been shown to have the ability to reduce the expression of the inflammatory genes TNFα, IL-6, IL-1β, and COX-2, by suppressing the activation of NF-κB and JNK in primary cultures of human adipocytes and macrophages [141]. This mechanism was confirmed in a recent in vitro study, in which quercetin and catechin, alone or in combination, were able to suppress significantly TNF-α and IL-1β genes and proteins expression in LPS-stimulated RAW 264.7 cells, through the inhibition of TLR4-mediated NF-κB p65/p50 and JNK phosphorylation [142]. In a recent in vivo study, quercetin supplementation has been observed to attenuate the HFD-induced adipose tissue expansion in both subcutaneous and visceral fat depots of obese mice, resulting in the appearance of a multilocular phenotype in iWAT, which may be indicative of the browning process. These changes in WAT morphology occur in association with reduced gene expression of inflammatory markers, including MCP-1, F4/80, Cd68 and Cd11b [143]. Furthermore, quercetin supplementation significantly suppressed the proinflammatory cytokines TNFα, IL-6, and MCP-1 levels in eWAT and sera, suggesting its ability to reduce obesity-induced adipose tissue and systemic inflammation. These effects were mediated by AMPKα1 activation and SIRT1 expression [144]. For the first time, quercetin showed browning effects in WAT of HFD-fed mice and 3T3-L1 cells, through the activation in part of AMPK/SIRT1/PGC1α pathway, resulting in induction of BAT-like genes [145]; while the thermogenic effect of quercetin in BAT was only investigated in a study published in October 2020 [146]. In this study, BAT of HFD-fed mice supplemented with 1% of quercetin for 16 weeks, showed a significantly increase of UCP1 gene and protein expression, partly due to the activation of AMPK/SIRT1/PGC1α pathway [146] (Table 1).

## 5. Conclusions

The obesity-associated chronic low-grade inflammation of adipose tissue provides the basis for the development of a myriad of metabolic disorders. The main source of this inflammation is WAT, an active endocrine organ secreting adipokines, which can directly interfere with insulin signaling and recruit macrophages contributing to an inflamed state in the adipose tissue. Only recently, it has been seen that in obesity, inflammatory mediators affect negatively BAT function, damaging its capacity for energy expenditure and glucose uptake, and browning process in WAT, contributing to obesity-associated metabolic disease. In this context, the clarification of how inflammatory mediators control intracellular pathways during the dysfunction of the two types of adipose tissue seems necessary for the development of novel therapeutic strategies. Among them, weight loss and lifestyle modification can be effective in relieving systemic and adipose tissue inflammation, although these approaches are difficult to maintain for a long time. On the other hand, the suppression of inflammation, through the use of pharmacological agents has shown unpleasant side-effects. Therefore, in this review we have focused our attention on the role of nutraceutical approach in management of adipose tissue inflammation; in particular, we have collected the most recent evidence in the literature on the effects of the main polyphenols, known for their antioxidant and anti-inflammatory activity, highlighting their potential effects on common mediators and inflammatory pathways, responsible for WAT and BAT dysfunction. Although further research is needed to demonstrate that targeting pro-inflammatory mediators improves adipose tissue dysfunction and activates thermogenesis in BAT and WAT browning in the obese state, polyphenols supplementation could represent an innovative therapeutic strategy to improve metabolic health and prevent progression of obesity and obesity-related metabolic diseases.

## Figures and Tables

**Table 1:** Schematic representation of Citrus polyphenols effects on inflammation and metabolic alterations associated with obesity.

Polyphenols	Properties	Molecular Mechanisms	References
**Resveratrol**	↑ fatty acid oxidation; ↓ hepatic gluconeogenesis; ↑ glucose uptake	↑ AMPK activation	[111,112]
	↓ adipose tissue inflammation	↓ NF-κB and ERK activation; ↓ TLR2 and TLR4 expression; ↑ adiponectin levels; ↓ inflammatory cytokines expression and secretion	[113,114,115]
	↑ WAT browning	↑ SIRT1 activation	[116,117,118]
	↓ intestinal inflammation; ↑ integrity of the intestinal barrier	Improvement of gut microbiota composistion	[119]
**Curcumin**	↓ adipose tissue inflammation	↓ NF-κB activation; ↓ ER stress; ↓ JNK activation ↑ SIRT1-mediated Foxo1 activation and Foxo1 and (C/EBPα) interaction	[121,122,123]
	↑ UCP1 in mouse BAT	↑ PPARα and PPARγ activation	[124]
**Naringenin and Hesperetin**	↓ TNFα-stimulated FFA secretion both in 3T3-L1 adipocytes and in mouse epididymal primary adipocytes	↓ NF-κB and ERK pathways	[130]
**Naringenin**	↓ adipose tissue inflammation	↑ PPARγ activation ↓ NF-κB and JNK pathways	[131,132]
**Bergamot Polyphenolic Fraction (BPF)**	lipid-lowering properties: ↑ fecal neutral sterols and the total excretion of bile acids; ↓ cholesterol absorption, via inhibition of pancreatic cholesterol ester hydrolase (pCEH); statin-like action		[133,134,135,136,137]
	anti-inflammatory and antioxidant activity in the liver	↓ JNK/p38 MAPK pathways	[138,139]
**Nobiletin**	↓ body and total WAT weight; ↓ plasma TGs;	↑ plasma adiponectin levels; ↑ mRNA levels of lipogenesis-related genes (PPARγ, FAS, SREBP-1c and SCD-1) in WAT; ↑ energy expenditure related genes (PPARα, CPT-1 and UCP-2) in WAT	[140]
	anti-inflammatory activity in WAT	↓ activation of IKK/NF-κB pathway	
	Improved insulin signaling in WAT	↑ Akt phosphorylation and GLUT4 expression	
**Quercetin**	↓ adipose tissue inflammation	↓ activation of NF-κB and JNK; ↑ AMPKα1 activation and SIRT1 expression	[141,142,144]
	↑ WAT browning; ↑ UCP1 in mouse BAT	↑ AMPK/SIRT1/PGC1α pathway	[145,146]

↑ = increase; ↓ =decrease
